# Thymine Sensitizes Gram-Negative Pathogens to Antibiotic Killing

**DOI:** 10.3389/fmicb.2021.622798

**Published:** 2021-01-28

**Authors:** Yuan Liu, Kangni Yang, Yuqian Jia, Jingru Shi, Ziwen Tong, Zhiqiang Wang

**Affiliations:** ^1^College of Veterinary Medicine, Yangzhou University, Yangzhou, China; ^2^Institute of Comparative Medicine, Yangzhou University, Yangzhou, China; ^3^Jiangsu Co-Innovation Center for Prevention and Control of Important Animal Infectious Diseases and Zoonoses, Yangzhou University, Yangzhou, China; ^4^Joint International Research Laboratory of Agriculture and Agri-Product Safety, The Ministry of Education of China, Yangzhou University, Yangzhou, China

**Keywords:** antibiotic tolerance, thymine, Gram-negative bacteria, metabolism, antibiotic resistance

## Abstract

Diminished antibiotic susceptibility of bacterial pathogens is an increasingly serious threat to human and animal health. Alternative strategies are required to combat antibiotic refractory bacteria. Bacterial metabolic state has been shown to play a critical role in its susceptibility to antibiotic killing. However, the adjuvant potential of nucleotides in combination with antibiotics to kill Gram-negative pathogens remains unknown. Herein, we found that thymine potentiated ciprofloxacin killing against both sensitive and resistant-*E. coli* in a growth phase-independent manner. Similar promotion effects were also observed for other bactericidal antibiotics, including ampicillin and kanamycin, in the fight against four kinds of Gram-negative bacteria. The mechanisms underlying this finding were that exogenous thymine could upregulate bacterial metabolism including increased TCA cycle and respiration, which thereby promote the production of ATP and ROS. Subsequently, metabolically inactive bacteria were converted to active bacteria and restored its susceptibility to antibiotic killing. In *Galleria mellonella* infection model, thymine effectively improved ciprofloxacin activity against *E. coli*. Taken together, our results demonstrated that thymine potentiates bactericidal antibiotics activity against Gram-negative pathogens through activating bacterial metabolism, providing a universal strategy to overcome Gram-negative pathogens.

## Introduction

Infectious diseases caused by Gram-positive and Gram-negative bacterial pathogens that are resistant to clinically relevant antibiotics constitute a worldwide public crisis (Boucher et al., 2009; Van Boeckel et al., 2019). According to the Centers for Disease Control and Prevention (CDC) report in 2019, more than 2.8 million infections by antibiotic-resistant bacteria occur in the U.S. each year, and result in more than 35,000 death (CDC., 2019). Compared with Gram-positive bacteria, Gram-negative bacteria are harder to treat owing to the highly impermeable outer membrane and limited availability of antibiotics in the clinical setting (Li et al., 2015; Domalaon et al., 2018; Liu et al., 2019a). Thus, there is urgent need to identify novel strategies to combat Gram-negative bacterial infections. In addition to resistance genes-mediated antibiotic resistance, phenotypic resistance mechanisms have been proposed to act a crucial and unappreciated role in the burden of bacterial infections particularly chronic and recurrent infections (Meylan et al., 2018; Stokes et al., 2019). Accordingly, this phenotypic resistance could occur in all bacteria, and commonly correlate with the decreased metabolic state of bacteria, including impaired tricarboxylic acid (TCA) cycle, bacterial respiration and bacterial proton motive force (PMF) (Bhargava and Collins, 2015; Stokes et al., 2019). For example, cytochrome oxidase null mutant (Δ*cyoA* Δ*cydB* Δ*appB*) was highly resistant to the lethal effects of bactericidal antibiotics (Lobritz et al., 2015), suggesting that the metabolic state of bacteria is linked to antibiotic efficacy.

By contrast, boosting bacterial metabolism has been shown to effectively improve antibiotic activity against multiple pathogens (Bhargava and Collins, 2015; Liu et al., 2019b, 2020d). For instance, genetically increasing the basal respiration rate of *E. coli* increased bactericidal antibiotic efficacy over wild-type cells (Lobritz et al., 2015). In addition, specific exogenous metabolites supplementation that can promote bacterial metabolisms were also found to enhance antibiotic efficacy. Supplementation of exogenous alanine and/or glucose significantly enhanced the bactericidal activity of aminoglycoside against spontaneous kanamycin-resistant *Edwardsiella tarda* via accelerating TCA cycle, the production of nicotinamide adenine dinucleotide (NADH) and proton motive force (PMF) (Peng et al., 2015). Glutamate potentiated aminoglycoside efficacy against *Escherichia coli* and *Edwardsiella tarda* through a previously unknown prevalent pathway called pyruvate cycle (the P cycle) (Su et al., 2018). Meanwhile, exogenous glucose increased gentamicin killing against drug-resistant *Vibrio alginolyticus* through promoting the P cycle, NADH and intracellular gentamicin accumulation (Zhang et al., 2019). Moreover, the addition of small thiols such as cysteine can prevent the formation of drug tolerant and drug-resistant cells in cultures (Vilcheze et al., 2017). These examples suggest that exogenous metabolites such as various amino acids have a great potential to restore bacterial susceptibility to antibiotic killing through altering the metabolic state of bacteria.

Nucleotides, one of the important metabolites in organisms, are widely distributed in the human body and have a variety of biological functions, including nucleic acid composition, energy storage, cell metabolism and physiological regulation (Rudolph, 1994; Roy et al., 2016). Recently, an integrated “white-box” biochemical screening and machine learning approach was developed for revealing causal mechanisms for antibiotic efficacy (Yang et al., 2019). This study showed that purine biosynthesis participates in antibiotic lethality and antibiotic-induced adenine limitation increased ATP demand, which in turn elevated central carbon metabolism and oxygen consumption, and consequently enhanced the bactericidal activity of antibiotics. Furthermore, decreased nucleotides such as adenosine was found in antibiotic-resistant bacteria by GC-MS-based metabolomic profiling (Peng et al., 2015) and elevated pyrimidine was associated with increasing kanamycin killing (Su et al., 2015). Given that the intrinsic correlation between purine biosynthesis and nucleotides, we reasoned that nucleotides may play a positive effect on bacterial metabolism, and further affect antibiotic efficacy.

To that end, we investigated the effect of five nucleotides on the bactericidal activity of antibiotics against Gram-negative bacteria. Consequently, we find that thymine could potentiate multiple bactericidal antibiotics activity against various Gram-negative pathogens through activating bacterial metabolism and respiration, triggering the production of reactive oxygen species (ROS) and thereby improving antibiotic efficacy. These findings suggest that thymine has great potential as a metabolic regulator in assisting antibiotics to eliminate Gram-negative bacteria.

## Results

### Thymine Potentiates Ciprofloxacin Killing in a Growth Phase-Independent Manner

**FIGURE 1:**
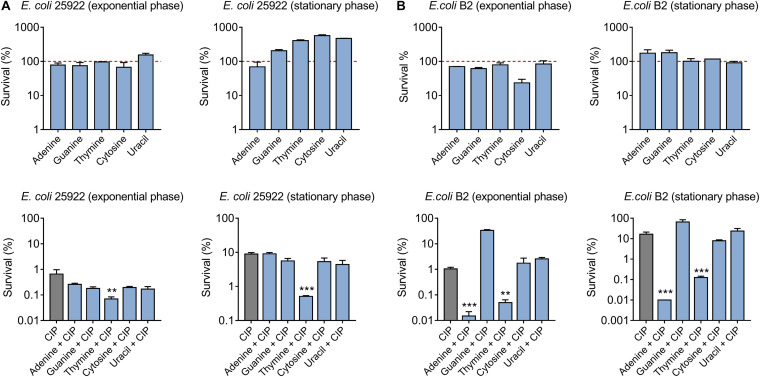
Thymine potentiates ciprofloxacin killing against both sensitive and resistant-*E. coli* in a growth phase-independent manner. **(A)** Effect of five nucleotides on ciprofloxacin activity against exponential and stationary phase *E. coli* ATCC 25922. **(B)** Adenine and thymine drastically improved ciprofloxacin killing against exponential and stationary phase MDR *E. coli* B2. All data were obtained in three independent experiments and shown as mean ± SD. *P < 0.05, **P < 0.01, ***P < 0.001, determined by one-way ANOVA.

To investigate the effect of nucleotides on antibiotic activity against Gram-negative bacteria, a standard sensitive *E. coli* ATCC 25922 and multidrug-resistant (MDR) clinical isolate *E. coli* B2 that is almost resistant to all available antibiotics, were chosen (Liu et al., 2020b; Song et al., 2020). Firstly, we assessed the ciprofloxacin killing (10-fold MIC) against different growth-phase bacteria through counting the surviving percentage of bacteria after co-treatment with five nucleotides. With respect to *E. coli* ATCC 25922, we found that five nucleotides alone had no bactericidal effect on exponential-phase bacteria, and in some extent promoted the stationary-phase bacterial growth. However, the combination of thymine and ciprofloxacin markedly decreased the survival rate of bacteria with above 1-log_10_ in a growth phase-independent manner (Figure 1A). Furthermore, a detailed time-killing curve of *E. coli* 25922 during 4 h treatment indicated that purines supplementation slightly undermined the killing activity of ciprofloxacin, whereas pyrimidines particularly thymine drastically potentiated ciprofloxacin activity (Supplementary Figure S1). As for *E. coli* B2, we found that adenine showed the best potentiation activity with ciprofloxacin with above 2-log_10_ against both exponential phase and stationary phase bacteria. In line with *E. coli* ATCC 25922, thymine also enhanced the ciprofloxacin killing against *E. coli* B2 (Figure 1B). In addition, we tested the synergistic activity of ciprofloxacin with five nucleotides against another reference strain *E. coli* MG1655. As a result, only thymine was found to improve ciprofloxacin activity against *E. coli* MG1655 (Supplementary Figure S3A), suggesting that thymine-induced potentiation to antibiotic is a general phenomenon across the species and adenine-mediated potentiation is specific to MDR *E. coli* B2.

**FIGURE 2:**
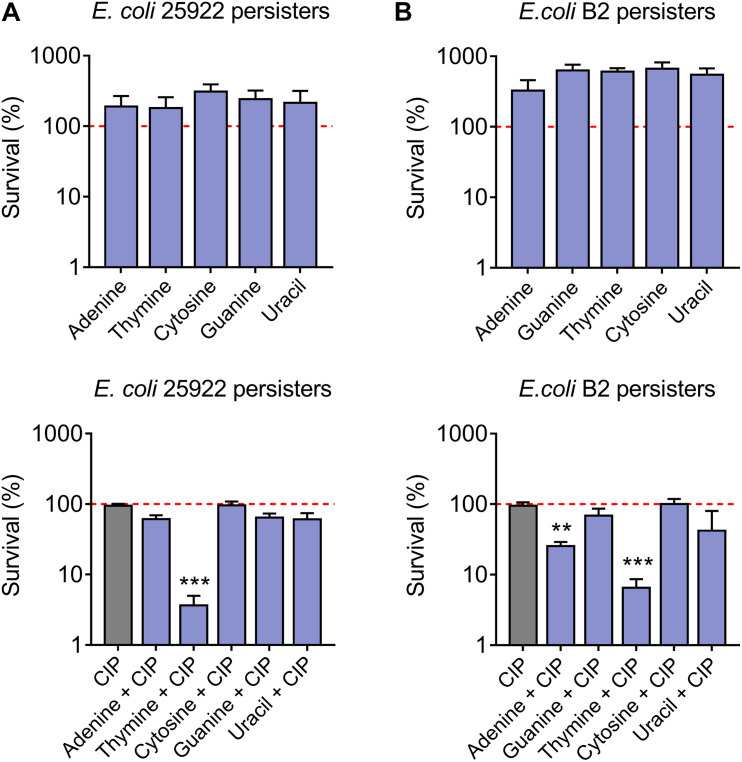
Synergistic bactericidal activity of thymine and ciprofloxacin against *E. coli* persisters. Percentage survival of *E. coli* 25922 persisters **(A)** and *E. coli* B2 persisters **(B)** after exposure to nucleotides (10 mM) or in combination with ciprofloxacin (20-fold MIC, 0.16 μg/mL and 320 μg/mL for *E. coli* 25922 and *E. coli* B2 persisters, respectively) for 4 h. All data were obtained in three independent experiments and shown as mean ± SD. *P < 0.05, **P < 0.01, ***P < 0.001, determined by one-way ANOVA.

Persister cells are a subpopulation of a clonal bacterial population that can survive in high concentrations of antibiotics (Fisher et al., 2017). To assess the synergistic bactericidal activity of nucleotides with antibiotics against persisters, *E. coli* 25922 (Figure 2A) and *E. coli* B2 persisters (Figure 2B) were treated with ciprofloxacin (20-fold MIC) in the presence or absence of 10 mM different nucleotides. As a result, thymine remarkably improved ciprofloxacin killing against persister cells, while five nucleotides alone triggered the growth of bacterial persisters (Figure 2). Taken together, these results indicated that adding nucleotides alone during bacterial growth may promote their reproduction, possibly because nucleotides are one of the indispensable substances for bacterial growth. By contrast, some nucleotides particularly thymine in combination with bactericidal antibiotic ciprofloxacin, results in enhanced killing effect against both sensitive and resistant-*E. coli* in a growth phase-independent manner.

### Thymine Enhances the Efficacy of Bactericidal Antibiotics Rather Than Bacteriostatic Antibiotics Against Gram-Negative Pathogens

Accordingly, bacteriostatic and bactericidal antibiotics result in two fundamentally different phenotypic outcomes, the inhibition of bacterial growth or cell death, respectively (Pankey and Sabath, 2004). They also have differences in perturbing bacterial metabolism. For example, bacterial growth inhibition result from bacteriostatic antibiotics is accompanied with suppressed cellular respiration, whereas cell death from most bactericidal antibiotics is correlated with enhanced respiration (Lobritz et al., 2015). To further validate the potentiating effect of thymine, we tested the survival rate of four kinds of Gram-negative pathogens including *E. coli* 25922, *S. enteritidis* 13076, *A. baumannii* 19606, and *P. aeruginosa* PA14 after combination treatment with bactericidal antibiotics (ampicillin and kanamycin) and bacteriostatic antibiotic such as tetracycline. Consequently, we found that thymine profoundly increased ampicillin and kanamycin activity against multiple pathogens, while no significant alteration for tetracycline (Supplementary Figure S2). These data demonstrated that thymine showed the universal potentiation with bactericidal rather than bacteriostatic antibiotics against a panel of Gram-negative pathogens.

### Thymine Sensitizes Gram-Negative Pathogens to Antibiotic Killing Through Upregulating Bacterial Metabolism

**FIGURE 3:**
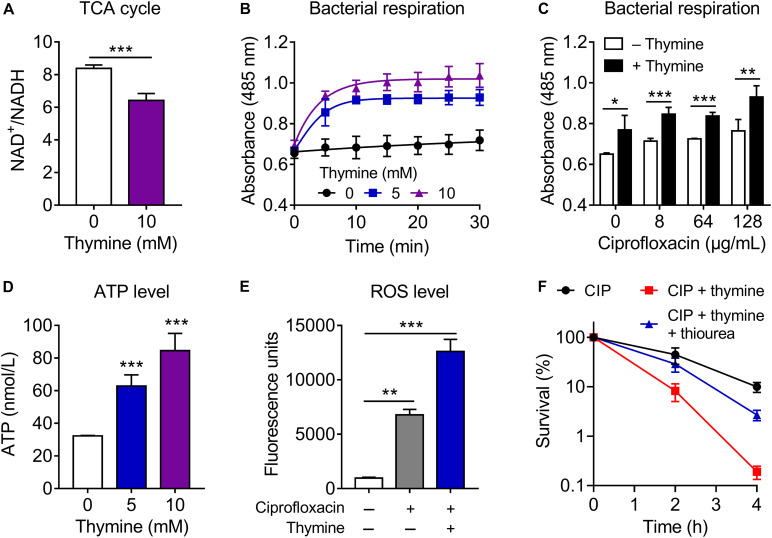
Thymine sensitizes Gram-negative pathogens to antibiotic killing through upregulating bacterial metabolism. **(A)** Thymine decreased the ratio of NAD^+^/NADH, measured by using the water-soluble tetrazolium salt WST-8. **(B,C)** Thymine significantly enhanced bacterial respiration in the absence **(B)** or presence **(C)** of ciprofloxacin through monitoring reduction of iodonitrotetrazolium chloride (INT). **(D)** Dose-dependent increase of intracellular ATP level by thymine. ATP level was determined based on bioluminescent reaction catalyzed by firefly luciferase. **(E)** Combination of ciprofloxacin and thymine enhanced ROS production, determined by 2′,7′-dichlorodihydrofluorescein diacetate (DCFH-DA, 10 μM). **(F)** Addition of ROS scavenger thiourea (100 mM) abolished the synergistic bactericidal activity of ciprofloxacin and thymine. All data were obtained in three independent experiments and shown as mean ± SD. *P < 0.05, **P < 0.01, ***P < 0.001, determined by unpaired *t*-test.

To investigate the underlying mechanisms of action of thymine in potentiating antibiotic efficacy, we firstly determined the MICs of antibiotics in the absence and presence of thymine. As a result, thymine had no effect of the MIC values of antibiotics against both *E. coli* ATCC 25922 and *E. coli* B2 (Supplementary Table S3). It has been suggested that antibiotic could target bacterial energy-consuming processes, which result in perturbation of bacterial metabolic homeostasis (Akhova and Tkachenko, 2014). Meanwhile, antibiotic efficacy could be correspondingly enhanced by altering the metabolic state of bacteria (Stokes et al., 2019). Since thymine could not decrease the MIC values of bactericidal antibiotic but still enhance antibiotic killing, we hypothesized that thymine may alter bacterial metabolism and sensitize Gram-negative bacteria to antibiotics. Several studies have been demonstrated that the TCA cycle is a shared mechanism in antibiotic lethality (Kohanski et al., 2007; Meylan et al., 2017). Therefore, we determined the effect of thymine on the bacterial TCA cycle through monitoring the ratio of NAD^+^/NADH. Interestingly, the addition of thymine (10 mM) significantly reduced the ratio in stationary-phase *E. coli* B2 (Figure 3A), indicating that thymine promoted the conversion of NAD^+^ to NADH and thereby possibly accelerated the TCA cycle. Similarly, a decreased ratio of NAD^+^/NADH in *E. coli* MG1655 in thymine-treated group was observed (Supplementary Figure S3B). To assess the role of the TCA cycle in the potentiation of thymine, we examined the cell viability in a TCA cycle gene knockout strain *E. coli* MG1655 (Δ*mdh*) after treatment with ciprofloxacin or in combination with nucleotides. As a consequence, we found that the potentiation of thymine was modestly abolished in knockout strain compared with wild type (Supplementary Figure S3C), implying the crucial role of TCA cycle in the potentiation of thymine.

Considering that the production of NADH would serve to regulate bacterial electron respiratory chain, thus we next assess whether thymine might elevate bacteria respiration. During 30 min monitoring, we found that stationary-phase *E. coli* B2 displayed weak respiration rate, whereas the thymine supplementation remarkedly enhanced bacterial respiration (Figure 3B). Meanwhile, the combination of ciprofloxacin and thymine could further improve bacterial respiration compared with ciprofloxacin alone (Figure 3C). Consistent with our observation, it has been shown that enhanced respiration prevented drug tolerance and drug resistance in *Mycobacterium tuberculosis* and improved isoniazid killing (Vilcheze et al., 2017). In addition, we determined the intracellular ATP level after exposure to thymine. In agreement with the enhanced respiration, thymine significantly promoted the production of ATP (Figure 3D). This result also explains the profound potentiation of thymine with aminoglycoside antibiotics, which require transmembrane electrochemical gradient for enough cell penetration. These results indicated that the addition of thymine stimulates bacterial respiration and converts tolerant cells to metabolically active cells. Consequently, active bacterial cells would be more sensitive to bactericidal antibiotic killing.

Despite some controversy, ROS has been shown to be critical for antibiotic killing. Although bactericidal antibiotics have well-established mechanisms of action, recent studies revealed that ROS generation is the common consequence of the interaction of antibiotics with their classical targets (Zhao and Drlica, 2014; Acker and Coenye, 2017), which eventually impair synthesis of DNA, lipids and protein. It is well-accepted that the majority of superoxide generation in *E. coli* occurs through oxidation of the respiratory electron transport chain driven by oxygen (Kohanski et al., 2007). Therefore, we determined the ROS production in *E. coli* B2 after exposure to ciprofloxacin or in combination with thymine. Meaningfully, we observed enhanced ROS production in combinational treatment compared with ciprofloxacin alone (Figure 3E), which is in agreement with the finding that enhanced ciprofloxacin killing in the presence of thymine. Consistently, the addition of ROS scavenger thiourea markedly abolished the synergistic bactericidal activity of ciprofloxacin and thymine (Figure 3F), suggesting that ROS production is of importance for potentiation of thymine. Meanwhile, we evaluated pyruvate cycle ([TCA cycle plus phosphoenolpyruvate (PEP)-pyruvate-AcCoA pathway)], antioxidant and electron transport chain (ETC) related gene expression of *E. coli* B2 after treatment with increasing concentrations of thymine (Supplementary Figure S4). Dose-dependent increase of the expression of pyruvate cycle genes including *icd* (isocitrate dehydrogenase), *sucA* (2-oxoglutarate dehydrogenase), *mdh* (malate dehydrogenase), *fumA* (fumarase), *pck* (phosphoenolpyruvate carboxykinase), and *pykA* (pyruvate kinase II) was observed. In contrast, antioxidant genes such as *sodB* (superoxide dismutase) and *aphC* (alkyl hydroperoxide reductase) were remarkably decreased under treatment of thymine. Consistent with enhanced ATP levels by thymine, the expression of ETC associated genes including *nuoA* (NADH-quinone oxidoreductase), *cydA* (cytochrome oxidase), and *aptD* (ATP synthase) were significantly increased. Taken together, these results indicated that exogenous thymine could upregulate bacterial metabolism including increased TCA cycle and respiration, which thereby promote the production of ATP and ROS, and eventually sensitize Gram-negative pathogens to antibiotic killing.

### Thymine Supplementation Improves Ciprofloxacin Efficacy *in vivo*

**FIGURE 4:**
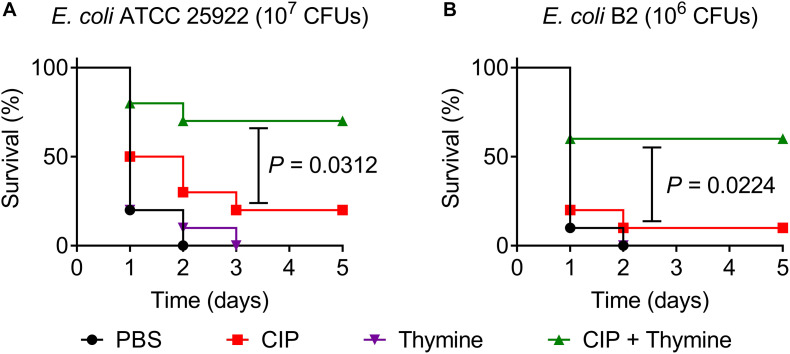
Thymine restores ciprofloxacin activity in *Galleria mellonella* infection model. *Galleria mellonella* larvae (*n* = 10 per group) was given a lethal inoculum of *E. coli* ATCC 25922 (10^7^ CFUs, **A**) or *E. coli* B2 (10^6^ CFUs, **B**). After 1-h post-infection, a single dose of PBS, ciprofloxacin (0.08 mg/kg for 25922 or 50 mg/kg for B2), thymine (10 mg/kg) and their combination were given. Survival rates of *Galleria mellonella* were monitored during 5 days. *P*-values were determined by the log rank (Mantel-Cox) test.

Given the attractive potentiation of thymine to bactericidal antibiotics *in vitro*, we next assessed its synergistic efficacy with ciprofloxacin in a *Galleria mellonella* infection model (Liu et al., 2020c; Pereira et al., 2020). *Galleria mellonella* larvae were infected with a lethal dose of *E. coli* ATCC 25922 or *E. coli* B2, then treated with a single dose of ciprofloxacin or thymine monotherapy or their combination. As shown in Figures 4A,B, all larvae after treatment with PBS or thymine died during 3 days, suggesting that thymine monotherapy has no therapeutic effect. Meanwhile, ciprofloxacin alone showed a weak effect on survival of larvae (only 20% for 25922 and 10% for B2). By contrast, combination therapy of ciprofloxacin and thymine significantly increased survival rate of larvae (above 50%) at 5 days following infection by *E. coli* ATCC 25922 (*P* = 0.0312) or *E. coli* B2 (*P* = 0.0224).

## Discussion

The growing infections caused by antibiotic resistant bacteria, coupled with a decreasing pipeline of new antibiotics pose a global public health crisis. As such, novel strategies that bypass the need for discovery or design of novel drugs are urgently needed to confront antibiotic resistance crisis. There is increasing evidence that the metabolic state of bacteria has an effect on their susceptibility to antibiotic, thus we proposed that antibiotic efficacy may be potentiated by activating bacterial metabolism with exogenous substances (Bhargava and Collins, 2015). Compared with traditional development of antibiotic adjuvants that target specific resistance elements or host immune response, modulating metabolic activity of bacteria offers a generalizable approach to improve bactericidal activity of antibiotics. In particular, such a strategy was first applied to improve aminoglycosides activity against bacterial persisters (Allison et al., 2011). In their study, Allison et al. (2011) showed that the combination of gentamicin and upper glycolysis-related metabolites such as glycose, mannitol and fructose displayed a better eradication of persisters with a CFU reduction of 3-log than gentamicin alone through increasing proton-motive force (PMF) and promoting aminoglycoside uptake. Furthermore, another study also showed that *L*-lysine potentiates aminoglycosides against *A. baumannii* via the same mechanisms (Deng et al., 2020). These works revealed that the supplementation of specific metabolites may modulate the metabolic state of bacteria and effectively potentiate their susceptibility to bactericidal antibiotics.

Although nucleotides are recognized as one of indispensable metabolites in all organisms, the underlying role of nucleotides in modulating antibiotic efficacy is yet unknown. Thus, in this study, we investigated the effect of five commonly used nucleotides on antibiotic killing against multiple Gram-negative bacteria. As a result, we found that thymine effectively potentiated bactericidal antibiotics killing against both exponential and stationary phase Gram-negative bacteria, as well as persisters and MDR clinical isolates. In addition, adenine only enhanced ciprofloxacin activity on MDR *E. coli* B2, but not reference strains *E. coli* ATCC 25922 and MG1655, suggesting that its potentiation is specific to resistant strain *E. coli* B2. We speculated that the supplementation of adenine may be related to the suppression of specific resistance determinants, however, the accurate modes of action warrants more in-depth investigation. Considering that thymine displayed a universal potentiating effect on various bactericidal antibiotics against multiple Gram-negative bacteria, we hypothesized that thymine may alter the metabolic state of bacteria. To test this, bacterial metabolism-related factors including TCA cycle, respiration, the production of ROS and ATP were measured. Consequently, we first observed a decrease in NAD^+^/NADH and an increase in respiratory rate of *E. coli* in the presence of thymine, which implies the acceleration of TCA cycle. Then, we found thymine supplementation remarkably enhanced bacterial respiration. Acceleration of the TCA and respiratory rate indicated an increase in the metabolic state of the bacteria, which was also evidenced by the altered ATP level by thymine. It has been acknowledged that the majority of ROS generation in bacteria comes from the oxygen-driven oxidation of the respiratory electron transport chain (Kohanski et al., 2007). Consistently, enhanced ROS production in the combination treatment was found compared with ciprofloxacin alone. ROS has been shown to be a common mechanism of cellular death induced by bactericidal antibiotics (Zhao and Drlica, 2014). For instance, *L*-serine potentiated ofloxacin and moxifloxacin to kill *E. coli* by increasing the production of NADH, disrupting the Fe-S clusters and thus stimulating endogenous ROS production (Duan et al., 2016). Exogenous alanine or glucose facilitated aminoglycosides killing of antibiotic-resistant *E. tarda* and *V. alginolyticus*, respectively, via promoting the generation of ROS (Ye et al., 2018; Zhang et al., 2020). These evidences suggest the importance of ROS in the metabolites-enabled potentiation to bactericidal antibiotics.

In summary, our data revealed the huge potential of thymine as a promising metabolism regulator for treatment of bacterial infections caused by Gram-negative bacteria. Specifically, thymine accelerates the bacterial metabolisms particularly respiration, triggers the production of ROS and thereby improves antibiotic efficacy both *in vitro* and *in vivo*. These finding inspired that enhancing bacterial metabolism may lead to the discovery of new treatments and improve current antibacterial therapies. However, more studies are still required to systematically assess the *in vivo* therapeutic potential of the combination of thymine and antibiotics in the clinical setting.

## Materials and Methods

### Bacterial Strains and Chemical Reagents

Five reference stain *Escherichia coli* ATCC 25922, *Escherichia coli* MG1655, *Salmonella enteritidis* ATCC 13076, *Acinetobacter baumannii* ATCC 19609, *Pseudomonas aeruginosa* PA14, and a multidrug-resistant (MDR) clinical isolate *Escherichia coli* B2 (Liu et al., 2019c, 2020b; Song et al., 2020) were used in this study. *Escherichia coli* MG1655 deleted mutant (Δ*mdh*) was obtained by homologous recombination mediated by suicide plasmid pLP12. Unless specifically noted, bacteria were grown in Mueller-Hinton broth (MHB) at 37°C with 200 rpm. To obtain different growth phase bacteria, overnight *E. coli* were diluted 1:1,000 into 5 mL fresh MHB, and grown to exponential phase (4 h) or stationary phase (8 h). To obtain bacterial persisters, *E. coli* B2 were grown to late-stationary phase for 16 h at 37°C and then pretreated with ciprofloxacin (20-fold MIC, 320 μg/mL) for 4 h to kill non-persistent cells (Morones-Ramirez et al., 2013). Surviving cells were resuspended in MHB, and confirmed as persiters by exposure to higher concentration of ciprofloxacin (40-fold MIC, 640 μg/mL) for 4 h and no further CFU reduction was observed. Ciprofloxacin, ampicillin, kanamycin and tetracycline were obtained from China Institute of Veterinary Drug Control. Nucleotides were purchased from TCI chemicals (Shanghai, China).

### MIC Measurements

MICs of different antibiotics were determined using the twofold serially microdilution method according to the Clinical and Laboratory Standards Institute. (2018) guideline (In, 2018). Then, the samples were incubated at 37°C for 18 h and the MIC values were interpreted as the lowest concentrations of antibiotics with no visible growth of bacteria. The MICs of each antibiotic for different bacteria have been shown in Supplementary Table S1.

### Bactericidal Activity of Antibiotics With or Without Nucleotides

To assess the effect of different nucleotides on the bactericidal activity of ciprofloxacin against *E. coli* ATCC 25922 and *E. coli* B2, exponential phase or stationary phase cells were treated with either ciprofloxacin (10-fold MIC, 0.08 μg/mL for *E. coli* ATCC 25922 and 160 μg/mL for *E. coli* B2) or five nucleotides (10 mM) alone or their combination for 4 h. Then, 100 μL aliquots were removed, centrifuged at 10,000 g for 5 min and resuspended in 100 μL PBS. Suspensions were serially diluted and spot-plated onto MH agar plates at 37°C overnight to determine CFUs/mL, and the percentage of surviving cells were calculated. In ROS quenching experiments, stationary phase*-E. coli* B2 were treated with ciprofloxacin, thymine and thiourea (100 mM) simultaneously. For *E. coli* 25922 and B2 pesisteres, 20-fold MIC of ciprofloxacin (0.16 μg/mL and 320 μg/mL for *E. coli* ATCC 25922 and *E. coli* B2 persisters) and/or five nucleotides (10 mM) were used. For the synergistic activity of ciprofloxacin and five nucleotides against stationary phase *E. coli* MG1655 or *E. coli* MG1655 (Δ*mdh*), the synergistic effect of thymine and other three antibiotics (ampicillin, kanamycin and tetracycline) against other three kinds of Gram-negative pathogens (*Salmonella enteritidis* ATCC13076, *Acinetobacter baumannii* 19606 and *Pseudomonas aeruginosa* PA14) in stationary phase, similar protocols were performed as described above.

### NAD^+^/NADH and Bacterial Respiration Determination

*E. coli* B2 were collected and diluted into OD_600_ = 0.5 in M9. Then, different concentrations of ciprofloxacin (0, 1, 5, and 10-fold MIC) were added with or without thymine (10 mM). After 4 h incubation, cell pellets were washed with 0.01 M PBS (pH = 7.2) and re-suspended with 200 μL precooled extraction buffer. The lysate was centrifuged at 12,000 g for 10 min at 4°C, and supernatant was measured using NAD^+^/NADH Assay Kit with WST-8 (Beyotime, China).

Effect of thymine on the bacterial respiration was monitored by reduction of iodonitrotetrazolium chloride (INT) (Wenzel et al., 2014). Briefly, *E. coli* B2 were diluted into OD_600_ = 0.5 in M9, and thymine (10 mM) was added. Subsequently, 1 mM INT and 0.6 mM NADH were added as substrate and the solution was incubated for 1 h in the dark. INT reduction was stopped by addition of 5% trichloroacetic acid. Insoluble formazan was centrifuged at 13,000 g for 5 min and extracted with 1 mL ethanol. Absorbance of the supernatant at 485 nm was measured during 60 min.

### ATP and ROS Measurement

Intracellular ATP levels of *E. coli* B2 were determined using an Enhanced ATP Assay Kit (Beyotime, China). *E. coli* B2 suspension was incubated with thymine (0, 5, and 10 mM) for 1 h. After incubation, bacterial cells were harvested and lysed with lysozyme, and the supernatant was prepared for ATP levels measurement. Detecting solution was added to a 96-well plate and incubated at room temperature for 5 min. Subsequently, the supernatants were added to the wells and its luminescence was measured by Infinite M200 Microplate reader (Tecan). Total ATP levels were calculated from the luminescence signals accordingly.

2′,7′-dichlorodihydrofluorescein diacetate (DCFH-DA, 10 μM) were added into *E. coli* B2 suspension (Liu et al., 2020a). After incubated at 37°C for 30 min, 190 μL of probe-labeled bacterial cells were added to a 96-well plate and 10 μL of ciprofloxacin (0, 1, 5, and 10-fold MIC) without or with thymine (10 mM) were added. After incubation at 37°C for 1 h, fluorescence units were immediately measured with the excitation wavelength at 488 nm and emission wavelength at 525 nm using an Infinite M200 Microplate reader (Tecan).

### Quantitative RT-PCR Analysis

Exponential phase *E. coli* B2 were incubated with thymine (0–10 mM) for 4 h. Total RNA was extracted reverse transcribed to cDNA using the PrimeScript^TM^ RT reagent Kit with gDNA Eraser (Takara). RT-PCR analysis was performed on a 7500 Fast Real-Time PCR System (Applied Biosystem, CA, United States) using the TB Green qPCR Kit (Takara) with the optimized primers (Supplementary Table S2). Relative quantitative method was applied to calculated the fold changes of mRNA expression relative to the housekeeping gene *rpoC* (for: GTACGTTCCACATCGGTGGT; *rev*: CAACCGACTTCACGTTGCTG) (Yang et al., 2020).

### *Galleria mellonella* Infection Model

*Galleria mellonella* larvae (purchased from Huiyude Biotech, Tianjin, China) were randomly divided into four groups (*n* = 10 per group) and infected with 10 μL of *E. coli* ATCC 25922 (10^7^ CFUs) or *E. coli* B2 suspension (10^6^ CFUs) at the right posterior gastropoda. After 1-h post-infection, larvae were treated with PBS, ciprofloxacin (0.08 mg/kg for 25922 or 50 mg/kg for B2), thymine (10 mg/kg), or the combination of ciprofloxacin with thymine at the left posterior gastropoda. Survival rates of *Galleria mellonella* were recorded during 5 days.

## Data Availability Statement

The raw data supporting the conclusions of this article will be made available by the authors, without undue reservation.

## Author Contributions

ZW and YL designed this study. YL and KY performed all experiments and wrote the manuscript. YL, KY, YJ, JS, and ZT analyzed the data. All authors have read and agreed to the published version of the manuscript.

## Conflict of Interest

The authors declare that the research was conducted in the absence of any commercial or financial relationships that could be construed as a potential conflict of interest.
