# Supplementary material for: Thymine Sensitizes Gram-Negative Pathogens to Antibiotic Killing
Source: Front Microbiol. 2021 Jan 28;12:622798. doi: 10.3389/fmicb.2021.622798 (PMC7875874; doi:10.3389/fmicb.2021.622798)
Supplement: Supplementary file 1 [file Data_Sheet_1.docx]

**Supplementary materials**

**Tables**

**Tables S1 Antimicrobial susceptibility of antibiotics against Gram-negative bacteria (MIC, μg/mL).**

| Organism | Ciprofloxacin | Ampicillin | Kanamycin | Tetracycline |
| --- | --- | --- | --- | --- |
| *E. coli* ATCC 25922 | 0.008 | 8 | 4 | 2 |
| *E. coli* B2 | 16 | >128 | >128 | 128 |
| *S. enteritidis* ATCC 13076 | - | 64 | 4 | 4 |
| *A. baumannii* ATCC 19609 | - | 0.5 | 4 | 4 |
| *P. aeruginosa* PA14 | - | 256 | 256 | 256 |

**Table S2 PR-PCR primers used in this study.**

| Genes | Sequence (5’→3’) | Product (bp) |
| --- | --- | --- |
| *icd* | CCCGAACACTGGCAAAGAGA | 138 |
|  | GCCAGGGCGTCAGAAATGTA |  |
| *sucA* | GCAGTTTGCTCACGTCAAGG | 90 |
|  | TCACGGAAATGATGCTGGCT |  |
| *mdh* | CGGTTATTGGCGGTCACTCT | 111 |
|  | CGTTCTGGATGCGTTTGGTC |  |
| *fumA* | TACTTCGCTCACGACATCCG | 118 |
|  | CCTGACGGTTGATCTTCGCT |  |
| *pck* | TATCGTTATGAACGGCGCGA | 79 |
|  | AAACGCCACGAAGTTTTCGG |  |
| *pykA* | CAAAACTGGGGCGTCATGTG | 143 |
|  | GTCGCCTTCACCTTTACCCA |  |
| *sodB* | TACGGCAAGCACCATCAGAC | 131 |
|  | ACCTGAGCTGCGTTGTTGAA |  |
| *ahpC* | GGCAATCGAAGTTACCGCTG | 148 |
|  | CAGAGACGGAGCCAGAGTTG |  |
| *nuoA* | AGTCATCGCTCATCACTGGG | 96 |
|  | CGACCGCCTAAAAACCAACC |  |
| *cydA* | GCGCTCTCTTTCTGGAGTGT | 101 |
|  | TCTACAGCAATCCACGGCAG |  |
| *atpD* | ACCACGAAATGACCGACTCC | 145 |
|  | AGAACGTCACGACCTTCGTC |  |

**Tables S3 MIC of antibiotics against *E. coli* ATCC 25922 and *E. coli* B2 with or**

**without thymine.**

| Strains | MIC (μg/mL) | | | | |
| --- | --- | --- | --- | --- | --- |
|  | Ampicillin | Kanamycin | Ciprofloxacin | Meropenem | Colistin |
| *E. coli* ATCC 25922 | 8 | 4 | 0.008 | 0.03 | 0.5 |
| *+* thymine (10 mM) | 8 | 4 | 0.008 | 0.03 | 0.5 |
| *E. coli* B2 | >128 | >128 | 16 | 32 | 8 |
| *+* thymine (10 mM) | >128 | >128 | 16 | 32 | 8 |

**Figures**





**Figure S1 Time-killing curve of *E. coli* ATCC 25922 by ciprofloxacin and/or** **nucleotides.**

Exponential phase *E. coli* ATCC 25922 was treated with ciprofloxacin (10-fold MIC) or the combination of ciprofloxacin and five nucleotides (10 mM) for 4 h. Bacterial loads were determined by plate count method at every 1 h.





**Figure S2 Percent survival of four reference Gram-negative bacterial cells in stationary phase after exposing to three antibiotics or in combination with thymine for 4 h.**

AMP, ampicillin; KAN, kanamycin; TET, tetracycline. Data from three independent experiments are shown as mean ± SD, and the difference compared with antibiotics alone were determined by unpaired *t* test (***P* < 0.01, ****P* < 0.001).





**Figure S3 Thymine potentiates ciprofloxacin activity against** ***E. coli* MG1655.**

**(A and C)** Survival of *E. coli* MG1655 (A) and its gene knockout mutant (Δ*mdh*, C) after treatment with ciprofloxacin (10-fold MIC) or in combination with five nucleotides (10 mM) for 4 h. Data were presented as mean ± SD, and the difference was determined by determined by one-way ANOVA (**P* < 0.05 and ***P* < 0.01).

**(B)** Thymine decreased the ratio of NAD^+^/NADH in *E. coli* MG1655. Difference was determined by determined by unpaired *t* test (***P* < 0.01).





**Figure S4 Thymine promotes the expression of pyruvate cycle and electron transport chain related genes, and inhibits the expression of antioxidant genes by RT-PCR analysis.**

All data were presented as mean ± SD (n = 3), and the difference was determined by determined by one-way ANOVA (**P* < 0.05, ***P* < 0.01, ****P* < 0.001).
